# The Effects of Economic Crisis on Health of the Serbian Population: What Do We Know So Far?

**Published:** 2019-02

**Authors:** Natasa POPOVIC, Zorica TERZIC-SUPIC, Milos ERIC, Jelena MARINKOVIC, Snezana SIMIC

**Affiliations:** 1. Institute of Social Medicine, Faculty of Medicine, University of Belgrade, Belgrade, Serbia; 2. Faculty of Economics, Finance and Administration, Singidunum University, Belgrade, Serbia; 3. Institute of Medical Statistic and Informatics, Faculty of Medicine, University of Belgrade, Belgrade, Serbia

## Dear Editor-in-Chief

The global financial crisis that originated in developed countries in 2008 has been spreading to the developing world, middle and low-income countries, and threatening years of progress on poverty reduction. Research community worldwide has agreed about challenges in studying the impact of economic crises on health, concerning the uniqueness of each crisis and investigating the lag effects, policies feedback and contradictory trends which made results ambiguous (1). Specificity of each crisis made its impact on public health different because of several key factors: the scale of the crisis, the nature of government responses, the pre-existing conditions and the extent to which population was exposed (1).

Adverse effects are seen to be concentrated among people experiencing or at risk of poverty, social exclusion, unemployment, and poor health and can be masked in the aggregate analysis, by improvements for other population (2). The adverse effects of economic crises on health and health system outcomes can be avoided or mitigated through public policy action (fiscal policy and social policy) (2). The government of the Republic of Serbia (RS) has responded to the crisis by adopting a package of measures based on a combination of monetary and fiscal policies (3). The consequences have manifested in reductions in government resources which generate fiscal pressure in the health system, which jeopardizes the financial sustainability of the Health Insurance Fund and capacity to fund the health care of vulnerable social groups (4). As a first response to the economic crisis in early 2009, the Ministry of Health (MOH) reviewed some of the policies which were flexible and sensitive enough to be adjusted to local conditions (5).

The study aims were to analyze trends in socioeconomic and health status indicators and to identify a possible association between economic recession and health of the Serbian population. The time trends analysis of socio-economic and health status indicators of the Serbian population (without data for Kosovo and Metohija), from 2000 to 2013, or 2012, was performed Four socio-economic indicators were included: real gross domestic product (GDP) expressed in purchasing power parity (PPP$) *per capita*, unemployment rate, total health expenditure as a percentage of GDP and total health expenditure (PPP$) *per capita*.

Morbidity indicators included are incidence rate (per 100000 inhabitants) of HIV, AIDS, TBC, cancer, cervix uteri cancer and female breast cancer. Mortality indicators included infant mortality rate and standardized cause-specific mortality rates (SDR) per 100000 inhabitants for diseases of circulatory system, cerebrovascular diseases, malignant diseases, diabetes mellitus, infections, and parasitic diseases and suicide. All indicators were taken from the European Health for All database.

Joinpoint regression analysis software was used to identify changes in trends of all indicators. The year 2008 was identified as a joinpoint for GDP *per capita* PPP$, the unemployment rate and total health expenditure (PPP$) per capita, demonstrating the onset of the global crisis on Serbian economy. The impact of the recession on health has raised significant concerns since 2008 with contradictory evidence about relationships between the current crisis and mortality and morbidity of the population. Our results indicate that most health indicators changed trends, although there was no statistically significant identification in 2008. In 2009 SDR for malignant and cerebrovascular diseases, showed a statistically significant decreasing trend (Table 1).

**Table 1:** Socio-economic indicators, health expenditure indicators and health status indicators, trends changes selected indicators

***Socio-economic indicators and health expenditure indicators***	***Joinpoint regression analysis***	
	***Period***	***APC (95%CI)***
Real gross domestic product, PPP $ per capita	2008–2013	2.3 (1.2 to3.5)^*^
Unemployment rate, %	2008–2012	9.9 (3.3 to16.9)^*^
Total health expenditure, PPP$ per capita, WHO estimates	2008–2013	−1.6 (−5.7 to 2.7)
**Health status indicators**		
*Morbidity based indicators: incidences rates per 100 000 inhabitants for:*		
AIDS	2008–2011	11.4 (−11.1 to 39.6)
	2011–2013	−11.8 (−29.6 to 10.5)
Cervix uteri cancer#	2009–2012	−2.0 (−10.7 to 7.5)
Female breast cancer #	2009–2012	1.3 (−11.0 to 15.3)
Mortality based indicator:		
*Standardized mortality rates per 100 000 inhabitants for:*		
Cerebrovascular diseases	2009–2013	−6.8 (−9.2 to −4.4)^*^
Malignant diseases	2009–2013	−1.1 (−2.0 to −0.3)^*^
Infectious and parasitic diseases	2009–2013	5.0 (−3.4 to 14.1)

* *P*<0.05

The possible explanation for variation could be the fact that various national health programs and strategies targeting to strengthen health promotion and prevention, implemented during the onset of the crisis, potentially contributed that some trends of health indicators of the population are not consistent with results of other studies (5). However, these policies were not represented as being direct responses to the crisis; they represent general policy trends in this area that were either partially or possibly mitigated the effects of the economic crisis. Conversely, adverse effects of the recession on health are likely to be expected, due to the long duration of the crisis, increasing poverty and the slow economic recovery.
